# Toll-Like Receptor 4 in the Rat Caudal Medulla Mediates Tooth Pulp Inflammatory Pain

**DOI:** 10.3389/fnins.2020.00643

**Published:** 2020-06-23

**Authors:** Helena F. Filippini, Graziella R. Molska, Maryam Zanjir, Yamini Arudchelvan, Siew-Ging Gong, Maria M. Campos, Limor Avivi-Arber, Barry J. Sessle

**Affiliations:** ^1^Programa de Pós-graduação em Odontologia, Escola de Ciência da Saúde e da Vida, Pontifícia Universidade Católica do Rio Grande do Sul, Porto Alegre, Brazil; ^2^Faculty of Dentistry, University of Toronto, Toronto, ON, Canada; ^3^Centro de Pesquisa em Toxicologia e Farmacologia, Escola de Ciências da Saúde e da Vida, Pontifícia Universidade Católica do Rio Grande do Sul, Porto Alegre, Brazil; ^4^Centre for the Study of Pain, University of Toronto, Toronto, ON, Canada; ^5^Departament of Physiology, Faculty of Medicine, University of Toronto, Toronto, ON, Canada

**Keywords:** mustard oil, tooth pulp inflammation, toll-like receptor – 4, electromyography, LPS, LPS-RS

## Abstract

The aims of this study were to investigate if Toll-like receptor 4 (TLR4) is expressed in the medullary dorsal horn (MDH) and if medullary application of a TLR4 antagonist (lipopolysaccharides from *Rhodobacter sphaeroides*, LPS-RS) can attenuate changes in nociceptive sensorimotor responses or TLR4 expression that might be evoked by mustard oil (MO) application to the right maxillary first molar tooth pulp. Of 41 adult male Sprague-Dawley rats used in the study, 23 received intrathecal application of the TLR4 antagonist LPS-RS (25 μg/10 μl; LPS-RS group) or isotonic saline (10 μl; vehicle control group) 10 min before pulpal application of MO (95%; 0.2 μl). Bilateral electromyographic (EMG) activities of the anterior digastric and masseter muscles were recorded continuously before and until 15 min after the MO application to the pulp. In 6 of these 23 rats and an additional 18 rats, the caudal medulla containing the ipsilateral and contralateral MDH was removed after euthanasia for subsequent Western Blot analysis of TLR4 expression in LPS-RS (*n* = 8) and vehicle (*n* = 8) groups and a naïve group (*n* = 8). The % change from baseline in the MO-evoked EMG activities within the anterior digastric muscles were significantly smaller in the LPS-RS group than the control group (two-way ANOVA, *post hoc* Bonferroni, *P* < 0.0001). Western Blot analysis revealed similar levels of TLR4 expression in the caudal medulla of the naïve, vehicle and LPS-RS groups. These novel findings suggest that TLR4 signaling in the caudal medulla may mediate MO-induced acute dental inflammatory pain in rats.

## Introduction

Tooth inflammatory pain is a common and debilitating condition with negative social and economic consequences that may result in an impaired quality of life if not appropriately treated. Regrettably, its underlying mechanisms are still not fully understood (Sessle, 2014; FDI World Dental Federation, 2015), and so studies aimed at enhancing understanding of its mechanisms are important for providing fundamental knowledge that could lead to improved management approaches to tooth inflammatory pain.

Several studies have shown that the medullary dorsal horn (MDH), which is often referred to as the trigeminal subnucleus caudalis, is an important caudal medullary site related to the processing and relaying of nociceptive information from the teeth and other orofacial structures to higher brain centers (for review, see Sessle, 1986, 2000, 2014; Byers and Närhi, 1999; Iwata et al., 1999, 2011; Chiang et al., 2011; Chichorro et al., 2017). For example, tooth pulp application of mustard oil (MO), an inflammatory irritant and transient receptor potential ankyrin 1 (TRPA1) agonist, can markedly enhance activity of MDH nociceptive neurons accompanied by nociceptive sensorimotor responses in jaw muscles reflected in MO-induced increases in electromyographic (EMG) activities (e.g., Chiang et al., 1998, 2007, 2011; Sunakawa et al., 1999; Narita et al., 2012). In addition, the MDH has been shown to be a critical interneuronal relay site in these reflex responses to MO and other noxious orofacial stimuli (Cairns et al., 1998, 2001; Tsai et al., 1999).

The neuronal hyperexcitability that can be induced in the MDH by MO or other noxious orofacial stimuli is considered to reflect a so-called central sensitization which involves the induction of neuroplastic changes in nociceptive processes in the central nervous system (CNS). In the trigeminal system, central sensitization has been documented to be an integral mechanism underlying acute and chronic pain states and to be dependent on the functional integrity of MDH microglia (Chiang et al., 2007, 2011; Iwata et al., 2011; Sessle, 2014; Chichorro et al., 2017). Microglial activation in pain states has been shown to involve the toll-like receptor 4 (TLR4) (Lehnardt et al., 2002; Olson and Miller, 2004; Guo and Schluesener, 2007; Kashima and Grueter, 2017; Bruno et al., 2018). TLR4 is a subtype of toll-like receptors involved in the recognition of lipopolysaccharides (LPS) present in the cell wall of gram-negative bacteria (Bryant et al., 2015; Gao and Li, 2016; Bruno et al., 2018). The stimulation of TLR4 results in the activation of two major intracellular signaling pathways: the myeloid differentiation primary response 88 (MyD88) dependent pathway and the TIR-domain-containing adapter-inducing interferon-b (TRIF) pathway. The first one induces nuclear factor-kB (NF-kB) translocation and expression of inflammatory cytokines as well as type I interferon genes, whereas the TRIF pathway activates type 1 interferon genes and delayed NF-kB translocation and expression of inflammatory cytokines via interferon regulatory factor 3 (IRF-3) (Vaure and Liu, 2014; Bruno et al., 2018).

TLR4 has been shown to be expressed in the spinal dorsal horn, the spinal analog of the MDH (Sun et al., 2015; Yan et al., 2015b; Hu et al., 2018). There is evidence that TLR4 may play a critical link between the innate and adaptive immune response as well as the induction, conversion, and maintenance of chronic pain states (Takeda et al., 2003; Bruno et al., 2018). Previous studies have documented the involvement of TLR4 processes in several pain models (Tanga et al., 2005; Christianson et al., 2011; Ohara et al., 2013; Tramullas et al., 2014; Sun et al., 2015; Bruno et al., 2018). Some of these studies have included investigations showing that TLR4 is involved in the induction phase of behavioral hypersensitivity in rodent pain models (Tanga et al., 2005) and that TLR4 antagonists such as LPS from *Rhodobacter sphaeroides* (LPS-RS) can attenuate nociceptive processes (Christianson et al., 2011; Sun et al., 2015). LPS-RS is a potent antagonist of toxic LPS in both human and murine cells, and also is effective in antagonizing effects attributed to TLR4 (Christianson et al., 2011; Sorge et al., 2011; Li et al., 2015; Sun et al., 2015). TLR4 expressed on microglia has been shown to contribute to spinal cord microglial activation and central sensitization (Lehnardt et al., 2002; Olson and Miller, 2004; Guo and Schluesener, 2007; Kashima and Grueter, 2017; Bruno et al., 2018). Microglial activation involving TLR4 processes leads to an increase of spinal inflammatory cytokines that maintain the proinflammatory environment within the spinal dorsal horn and thereby sustain central sensitization (Lehnardt et al., 2003; Tanga et al., 2005; Buchanan et al., 2010; Nicotra et al., 2012; Yan et al., 2015a; Bruno et al., 2018).

While the role of TLR4 in immune function, inflammation and spinal nociceptive mechanisms has been well established, knowledge of the role of this receptor in orofacial nociceptive processes is limited. It has been suggested that expression of TLR4 and its co-receptor CD14 in trigeminal sensory neurons may be related to the inflammatory pain resulting from tooth pulp infection (Wadachi and Hargreaves, 2006). LPS from gram-negative bacteria is the main exogenous TRL4 agonist during infection-associated dental pain and likely sensitizes the transient receptor potential vanilloid 1 (TRPV1) via TRL4 activation in the trigeminal sensory neurons (Diogenes et al., 2011; Green et al., 2016). There is also evidence that TRPV1 is co-expressed with TRPA1 in many sensory neurons, including those in tooth pulp, associated with small-diameter C-fibers in the trigeminal ganglion as well as in the dorsal root ganglion (Kobayashi et al., 2005; Sadofsky et al., 2014; Hargreaves and Ruparel, 2016; Gouin et al., 2017; Lee et al., 2019). This co-expression might lead to functional interactions between these two subtypes of TRP receptors (Fischer et al., 2014). However, although some studies (Ohara et al., 2013; Lin et al., 2015) have suggested the importance of TLR4 in orofacial pain states, the role of TLR4 in central mechanisms of dental pain is still unclear.

Some studies using pain models have shown increased expression of TLR4 in the spinal dorsal horn (Sun et al., 2015; Yan et al., 2015b; Hu et al., 2018). As noted above, the spinal dorsal horn is the spinal analog of the MDH, and earlier findings indicate that the MDH is a critical element in the neural circuitry underlying the reflex EMG activity that can be evoked in the jaw muscles by noxious orofacial stimuli. Therefore, the present study was initiated to use electrophysiological, pharmacological and molecular approaches to determine if TLR4 is expressed in the MDH and if medullary application of the TLR4 antagonist LPS-RS can attenuate these nociceptive sensorimotor responses or the increased TLR4 expression that might be evoked by MO stimulation of the rat tooth pulp.

## Materials and Methods

### Animals

**FIGURE 1:**
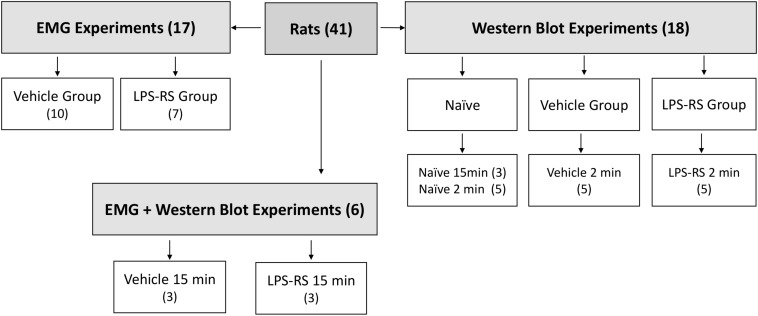
Schematic showing distribution of rats according to experimental groups. Note that some rats were solely used for the EMG experiments, some solely for the Western Blotting experiments, and some for both types of experiments. The EMG experiments included LPS-RS and vehicle groups of animals in which MO was applied to the tooth pulp after i.t. application of LPS-RS or vehicle, and the Western Blotting experiments involved LPS-RS and vehicle groups of animals euthanized at 2 and 15 min after MO application to the tooth pulp, as well as naïve animals.

A total of 41 adult male Sprague-Dawley rats (250–350 g) were obtained from Charles River (Montreal, QC, Canada). Social interaction and appropriate environmental conditions are important factors reducing anxiety and other emotions that can influence pain. Therefore, the rats were kept in their home cages (3 animals/cage) at the Department of Comparative Medicine (University of Toronto) under standard conditions of temperature (22 ± 2°C), light (12 h light-dark cycle) and humidity (50–70%) and provided with food and water *ad libitum*. The animals had an acclimatization time of 7 days before any experiment. All experimental procedures were carried out between 8:00 a.m. and 5:00 p.m. Twenty-three rats were used in the EMG experiments (6 of them were used in both the EMG and Western Blot experiments) and another 18 were used only in the Western Blot experiments described below. The sample sizes for the EMG experiments (*n* = 10–13 per group) and for Western Blot experiments (*n* = 3–5 per group) were established based on our previous studies documenting statistically significant findings in analogous experiments (Chiang et al., 1998, 2007; Sunakawa et al., 1999; Narita et al., 2012; Figure 1). All the procedures were approved by the Animal Care Committee of the University of Toronto (protocol number #20011420) and were accomplished in accordance with the regulations of the Ontario Animal Research Act (Canada).

### Drugs and Reagents

The inflammatory irritant and TRPA1 agonist mustard oil (MO – Allyl isothiocyanate, 95%; 0.2 μl, Aldrich-Sigma, CA, United States) was applied to the pulp of the right maxillary first molar to evoke EMG activities in the jaw muscles as previously described in detail (Chiang et al., 1998, 2007; Sunakawa et al., 1999; Narita et al., 2012). LPS from *Rhodobacter sphaeroides* (LPS-RS, InvivoGen, United States) was used as a TLR4 antagonist, as noted below. LPS-RS was dissolved in isotonic saline and it (or its vehicle – isotonic saline) was delivered by intrathecal (i.t.) administration to the caudal medulla overlying the MDH at a dose of 25 μg in a total volume of 10 μl, this dose of LPS-RS has been previously reported to be effective in attenuating mechanical allodynia when applied i.t. to L5/L6 in a spinal pain model (Sun et al., 2015). In preliminary experiments, Lipopolysaccharides (LPS) from *P. aeruginosa* (1 mg/ml and 5 mg/ml; Sigma, United States) (Shrestha et al., 2015) or from *E. coli* (10 mg/ml; Sigma, United States) (Renard et al., 2016) were applied to the pulp as a TLR4 agonist but were ineffective compared to MO in evoking EMG activities. Thus, the present study focused on the effects of the MO application to the pulp which has been well documented to induce jaw muscle EMG activity in rats (Sunakawa et al., 1999; Narita et al., 2012).

### General Experimental Protocols

Procedures followed our previously published standardized protocols (Avivi-Arber et al., 2010, 2015; Awamleh et al., 2015; Pun et al., 2016). For femoral vein cannulation, rats were anesthetized with intramuscular (i.m.) administration of ketamine (175 mg/kg) and xylazine (25 mg/kg), and then placed in a supine position. After cannulation, general anesthesia was maintained by intravenous (i.v.) administration of ketamine (75 mg/kg/h) for pulp exposure, insertion of EMG electrodes, neck dissection, and exposure of the caudal medulla. Subsequently, for EMG recordings, ketamine dosage was set at 25–50 mg/kg/h, and noxious pressure was periodically applied to the hind paw to ensure that it could induce a weak flexion reflex, indicating that an adequate and stable level of general anesthesia was obtained throughout the experimental sessions. Consistent with earlier electrophysiological recording and/or stimulation studies by ourselves and others, we used ketamine as a general anesthetic since, as compared with other general anesthetics (e.g., isoflurane), it does not suppress muscle tone (Nudo et al., 2003; Tennant et al., 2011, 2012; Avivi-Arber et al., 2015; Awamleh et al., 2015; Pun et al., 2016). A heating blanket (Model 73A, YSI, OH, United States) regulated by a rectal thermometer was used to maintain the animal’s body temperature (37–38°C), and a pulse oximeter monitored heart rate (333–430 beats/min) and oxygen saturation (90–100%).

### Pulp Exposure

The right maxillary first molar pulp was exposed using a low-speed dental drill with a round tungsten carbide bur (#1) under water cooling, as previously described (Chiang et al., 1998; Sunakawa et al., 1999; Narita et al., 2012). The pulp surface was covered with a piece of cotton wool soaked with isotone saline until the MO was applied to the pulp.

### EMG Electrode Insertion and Exposure of Medulla

EMG electrodes were made with 40-gauge, single-stranded, Teflon-insulated stainless- steel wires each with its final ∼5 mm end formed into a hook and a ∼1 mm exposed tip produced by stripping off its insulation (Cooner Wire, Chatsworth, CA, United States). After the pulp exposure, a pair of EMG electrodes (interpolar distance: ∼5 mm) was inserted into each of the left and right masseter muscles (LM; RM) and left and right anterior digastric muscles (LAD; RAD). Then the animal was turned and placed in a stereotaxic apparatus and the dorsal surface of the caudal medulla overlying the MDH was surgically exposed at the obex level for medullary application of drug or vehicle, as described below. The hooked end of each electrode provided a stable position of the electrodes in the muscle before, during and after the re-positioning of the animal. Furthermore, we confirmed the placement of EMG electrodes in each muscle and ensured that the muscle preparation had not deteriorated during the experiment. As previously described (Awamleh et al., 2015), this was achieved by delivering a constant-current stimulus (33.2 ms, 12 × 0.2 ms pulses, 333 Hz) to the muscle via the EMG electrodes and observing muscle twitches evoked in the muscle at a threshold stimulation intensity of ≤200 μA. This procedure was carried out immediately after EMG electrode placement, after positioning of the rat in the stereotaxic apparatus, and at the termination of the experiment.

### EMG Recordings

A rest period of 30 min was allowed after the surgery to ensure stable EMG recordings. A baseline level of EMG activities was then recorded for 15 min in all four jaw muscles (LM, RM, LAD, and RAD), and then continuously from baseline until 15 min after MO application to the pulp; 15 min was chosen since previous studies have shown the duration of EMG activity evoked by MO application to the tooth pulp is <20 min (e.g., Sunakawa et al., 1999; Narita et al., 2012). The EMG activity of each muscle was amplified (gain: 1000–5000; bandwidth: 30–3000 Hz) by an AC amplifier (A-M system, Washington, DC, United States, model 1700) and displayed on an oscilloscope, and was directly processed (i.e., rectified and integrated) by a computer interface 1401/program Spike 2 (CED, Cambridge, United Kingdom). In accordance with previous studies (Sunakawa et al., 1999; Narita et al., 2012), increases in EMG activity evoked by the application of MO were regarded as significant if one or more EMG area bins (mV/min) increased at least two standard deviations (SD) above the mean baseline level. To conserve on animal numbers, no naïve group or group with vehicle control applied to the tooth pulp or with tooth pulp exposure alone were used since we have previously shown that naïve animals or animals with vehicle (mineral oil) application to the pulp do not show evoked EMG (or MDH neuronal) activities (Chiang et al., 1998, 2007; Sunakawa et al., 1999; Narita et al., 2012). All EMG activities during the experimental session were measured and transformed into percentage values, by the division of the averaged baseline activity (mV/min) for the first 5 min of the 15 min baseline (Narita et al., 2012).

### Protocols of Treatment

LPS from *Rhodobacter sphaeroides* (LPS-RS) was selected based on previous studies of its effectiveness in antagonizing effects attributed to TLR4 (Christianson et al., 2011; Sorge et al., 2011; Li et al., 2015; Sun et al., 2015). The rats were divided into three groups (vehicle; LPS-RS; naive) (see Figure 1). To address LPS-RS effects on MO-evoked EMG activities, the LPS-RS group was pre-treated by i.t. application of LPS-RS (25 μg/10 μl) to the caudal medulla at 10 min before the pulpal application of MO. The vehicle group received the vehicle (isotonic saline) by the same i.t. application procedures as for the LPS-RS group. All i.t. injections were carried out under i.v. ketamine general anesthesia, as described above. The dura overlying the ipsilateral MDH (obex +0.5 mm, lateral 0.5 mm) was punctured with a 30-gauge needle attached to a Hamilton syringe positioned on the stereotaxic apparatus to permit the precise injection into the subdural space overlying MDH (Chiang et al., 1998; Narita et al., 2012; Takeda et al., 2013). Ten minutes after the pre-treatment with vehicle or LPS-RS, MO was applied to the exposed pulp. For this purpose, a small piece of dental paper point (diameter, 0.3 mm; length 0.5 mm) was soaked with MO (0.2 μl; 95%). The pulp cavity was then immediately sealed with temporary dental filling (Cavit, ESPE, Germany) to prevent any possible leakage of the chemical into other oral tissues.

### Blotting to Detect TLR4 Expression

For the Western Blotting experiments, we used LPS/RS, vehicle, and naïve groups of animals. A goal was to test if MO caused any change in TLR4 expression in MDH during and soon after the period of any MO-evoked EMG activity changes. We also wanted to determine if any suppressive effects of LPS-RS that might be demonstrated on MO-evoked jaw EMG activity was associated with any change in TLR4 expression in MDH, the site of the interneuronal relay of nociceptive inputs to trigeminal motoneurons (Cairns et al., 1998, 2001; Tsai et al., 1999). Since we found that the increased EMG activity persisted for a few minutes after MO application to the tooth pulp before returning to baseline levels (see section “Results”), the Western Blot analyses included LPS-RS group and vehicle group animals euthanized at either 2 min (LPS-RS group, *n* = 5; vehicle group, *n* = 5) or at 15 min (LPS-RS group, *n* = 3; vehicle group, *n* = 3) after application of MO to the pulp; naïve animals (*n* = 8) were also euthanized for the Western Blotting analysis (see Figure 1). The caudal medulla containing the ipsilateral and contralateral MDH was removed immediately after euthanasia for subsequent evaluation of TLR4 expression. The samples were homogenized on ice in 15 mmol/l Tris buffer containing a cocktail of proteinase and phosphatase inhibitors. The protein samples were separated via sodium dodecyl polyacrylamide gel electrophoresis (SDS–PAGE) and transferred onto polyvinylidene difluoride (PVDF) membranes. The membranes were placed in blocking buffer for 1 h at room temperature and incubated with a primary antibody against TLR4 (1:1000, Abcam, CA, United States) or Glyceraldehyde 3-phosphate dehydrogenase (GAPDH) (1:10,000, Cell Signaling, United States) overnight at 4°C. Then, the membranes were incubated in horseradish peroxidase-conjugated IgG (1:2000, Cell Signaling, United States). An enhanced chemiluminescence (ECL) solution (Pierce^TM^ Protein Biology, United States) was used to detect the immunocomplexes. Anti -GAPDH (Sigma-Aldrich, CA, United States) was used as loading control. The levels of TLR4 expression were quantified using GAPDH as an internal control as previously described (Sun et al., 2015).

### Statistical Analyses

The data are expressed as the mean ± the standard error. All EMG activities during the experimental session were measured and transformed into percentage values by the division of the averaged baseline activity (mV/min) recorded for the 5 min period prior to MO application in the experimental sessions. The data were normally distributed for RAD, LAD, RM, and LM in vehicle and LPS-RS groups, according to Agostino and Shapiro test or Kolmogorov-Smirnov test using GraphPad 8 software. A two-way analysis of variance (ANOVA), followed by Bonferroni test was used to test whether the independent variables (i.e., treatment with LPS-RS vs. vehicle and 1, 2, and 3 min time points after treatment) had any effect on the dependent variable (i.e., changes in EMG activities). For the Western Blotting analysis, data were evaluated by one-way ANOVA. *P*-values less than 0.05 were considered to indicate statistical significance.

## Results

**FIGURE 2:**
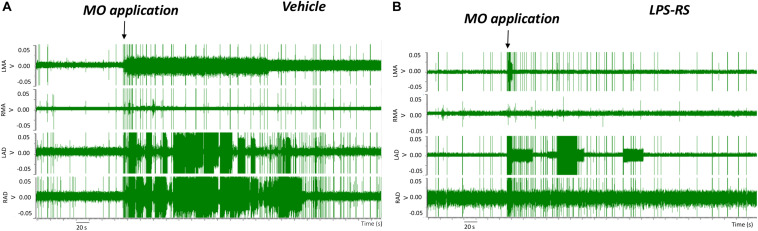
Examples of electromyographic (EMG) traces before and following mustard oil (MO) application to the first maxillary molar tooth pulp of rats treated 10 min earlier with i.t. application of either vehicle **(A)** or LPS-RS **(B)**. The four traces show the EMG activities (Volts, “V”) of the left masseter (LMA), right masseter (RMA), right anterior digastric (RAD), and left anterior digastric (LAD) muscles.

**FIGURE 3:**
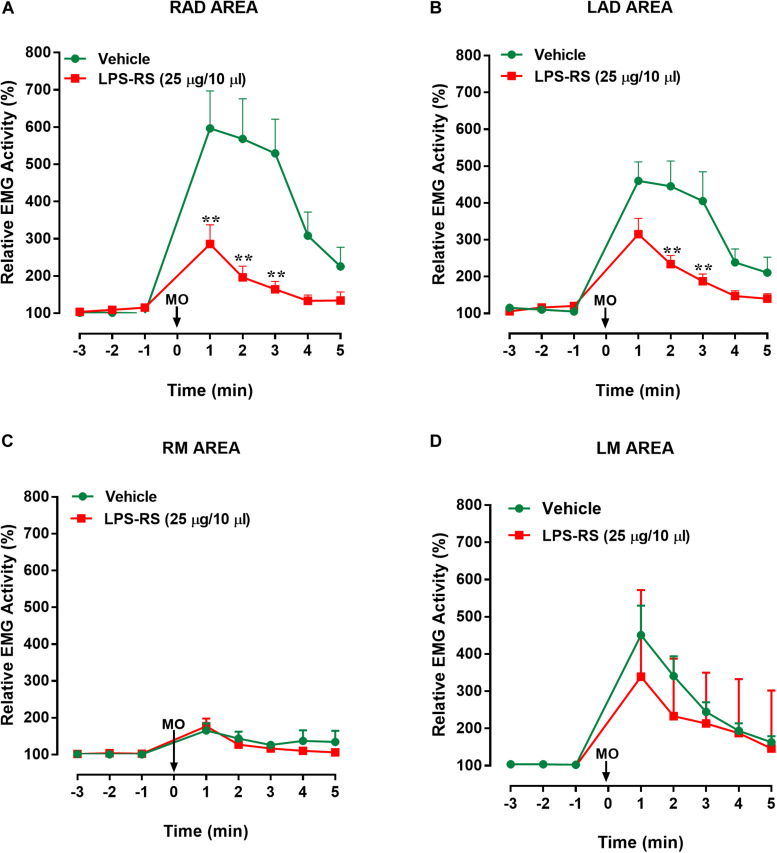
Effects of i.t. application of LPS-RS on the MO-evoked EMG activity expressed as % change from baseline, following the application of MO to the pulp of the right maxillary first molar. Data show the relative EMG activity changes (%) in **(A)** right anterior digastric (RAD), **(B)** left anterior digastric (LAD), **(C)** left masseter (LM), **(D)** right masseter (RM). The circles indicate data obtained in vehicle-treated animals, and the squares show the effects of LPS-RS (that was applied at 10 min before the MO application). Each point represents the mean ± standard error mean of 10–13 animals per group. ^∗∗^*P* < 0.0001 (two-way ANOVA followed by Bonferroni test).

EMG recordings in the control (vehicle) group revealed that MO application to the right maxillary molar tooth pulp resulted, within 2 s, in an increased EMG activity that was especially apparent in the LAD and RAD. The increased EMG activity persisted for up to 3 min before returning to baseline levels (Figures 2A, 3A,B). In contrast, in the LPS-RS group, i.t. application of LPS-RS 10 min prior to MO application to the tooth pulp, could attenuate the MO-evoked EMG responses (Figure 2B).

Statistical analysis revealed that there were statistically significant differences (*p* < 0.0001, 2-way ANOVA and *post hoc* Bonferroni) between the right and left masseter responses [vehicle group: *p* < 0.0001; *F*_(7, 161)_ = 9.88; LPS-RS group: *p* < 0.0001; *F*_(7, 126)_ = 5.93] and between right masseter and right anterior digastric responses, for both groups [vehicle group: *p* < 0.001; *F*_(7, 154)_ = 13.89; LPS-RS group: *p* < 0.0001; *F*_(7, 133)_ = 11.68]. In comparison with i.t. vehicle application, i.t. administration of LPS-RS could significantly attenuate the MO-evoked EMG responses only in the ipsilateral (RAD) and contralateral (LAD) anterior digastric muscles. In the RAD, decreased EMG activities were observed 1, 2, and 3 min following MO application to the tooth pulp [*p* < 0.0001; *F*_(7, 119)_ = 6.27] (Figure 3A). In the LAD, decreased EMG responses were observed only 2 and 3 min following MO application to the tooth pulp [*p* = 0.0002; *F*_(7, 147)_ = 23] (Figure 3B). No significant effects of LPS-RS were found in the masseter muscles (Figures 3C,D).

**FIGURE 4:**
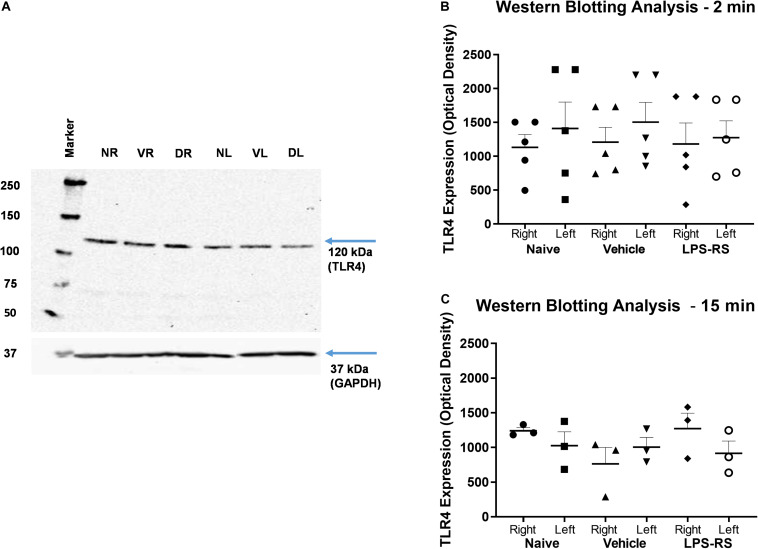
Representative Western Blot; TLR4 expression calculated in arbitrary units, considering GAPDH expression and naïve rats **(A)**. Western Blot analysis for TLR4 expression in the caudal medulla (right and left sides; *n* = 5 per LPS-RS group or vehicle group) at 2 min after MO application to the pulp **(B)**; Western Blot analysis for TLR4 expression in the caudal medulla (right and left sides; *n* = 3 per LPS-RS group or vehicle group) at 15 min after MO application to the pulp **(C)**. Western Blot analysis for TLR4 expression was also carried out in a total of 8 naïve animals. For **(A)**, NR, Naïve right side; NL, Naïve left side; VR, Vehicle right side; VL, Vehicle left side; DR, Drug (LPS-RS) right side; DL, Drug (LPS-RS) left side. For Western Blot analysis, the optical density values of each sample were used and evaluated by one-way ANOVA (*P* > 0.05).

Western Blot analysis showed expression of TLR4 in the right (ipsilateral) and left (contralateral) MDH of naïve rats and at 2 and 15 min following MO application to the tooth pulp in the LPS-RS and vehicle groups. There were no significant differences (*p* > 0.05) in the levels of TLR4 expression between the naïve, vehicle and LPS-RS groups (Figures 4A–C).

## Discussion

The present investigation has provided the first documentation that medullary (i.t.) pre-treatment with the TLR4 antagonist LPS-RS attenuates nociceptive sensorimotor responses in a rat model of acute dental inflammatory pain. The findings that the application of MO to the tooth pulp of the maxillary first molar in the vehicle group evoked a marked increase of jaw muscle EMG activities are consistent with previous studies reporting that MO application to the rat maxillary tooth pulp induces sensorimotor responses reflected in increased EMG activities in the jaw muscles (Sunakawa et al., 1999; Narita et al., 2012). It has been shown that naïve animals or animals receiving application of vehicle to the tooth pulp do not show increased jaw EMG activity or central sensitization in the MDH (e.g., Chiang et al., 1998; Sunakawa et al., 1999; Narita et al., 2012). Therefore, to conserve on animal numbers, we did not include control groups with tooth pulp exposure alone, or with vehicle (i.e., mineral oil) application to the tooth pulp. These earlier studies, as well as others (Hu et al., 1993; Lam et al., 2005), have also shown a differential magnitude of evoked responses between anterior digastric and masseter muscles or left and right muscles, and significant differences were noted in the present study between the right and left masseter responses and right masseter and right anterior digastric responses. Like the authors of the earlier studies, we cannot give any clear explanation for some of these differential patterns of EMG activity; future studies monitoring jaw movements together with EMG recordings in anesthetized as well as unanesthetized preparations may help in clarifying the basis for these EMG patterns and the movement patterns with which they are associated.

In addition, it has previously been shown that the increased jaw muscle EMG activities evoked by application of MO or other noxious stimuli to the pulp (or other orofacial tissues) have a reflex basis involving the activation of trigeminal pulp afferents and a relay via the MDH to the trigeminal motor nucleus where the trigeminal motoneurons supplying the jaw muscles are located (Yu et al., 1994; Cairns et al., 1998, 2001; Tsai et al., 1999; Sunakawa et al., 1999; Narita et al., 2012). Moreover, the short latency of the evoked EMG responses found in the present and previous studies suggest that they are initiated by direct excitatory action of MO on pulp afferents and a pauci-synaptic relay in brainstem (Sunakawa et al., 1999; Narita et al., 2012). The finding of increased EMG activities observed in the contralateral as well as ipsilateral anterior digastric muscles in the present study is in accordance with the findings of Cairns et al. (1998, 2001) and Tsai et al. (1999). These authors also revealed that the MDH plays an important role in the activation of contralateral as well as ipsilateral trigeminal motoneurons following the application of MO or other noxious stimuli to orofacial tissues.

Because the MDH is a critical element in neural pathways underlying the MO-evoked reflex responses (Cairns et al., 1998, 2001; Tsai et al., 1999), and TLR4 has been shown to be expressed in the spinal dorsal horn (Sun et al., 2015; Yan et al., 2015b) which is the spinal analog of MDH, the MDH was selected for i.t. administration of the TLR4 antagonist and for western blotting analysis. Our novel findings suggesting that TLR4 receptor processes in the rat caudal medulla may mediate nociceptive responses evoked by the TRPA1 agonist MO, which is a well-documented inflammatory irritant (Woolf and Wall, 1986; Hu et al., 1993; Yu et al., 1994; Cairns et al., 1998; Chiang et al., 1998), are consistent with previous studies documenting the presence of TRPA1 in the tooth pulp (Hargreaves and Ruparel, 2016; Lee et al., 2019) and the involvement of TLR4 and TRPA1 processes in several other pain models (Kobayashi et al., 2005; Christianson et al., 2011; Ohara et al., 2013; Fischer et al., 2014; Tramullas et al., 2014; Sun et al., 2015; Bruno et al., 2018). Some of these studies have included investigations showing that TLR4 antagonists such as LPS-RS can attenuate nociceptive processes (Christianson et al., 2011; Sun et al., 2015). LPS-RS is a potent LPS antagonist in murine and human cells and prevents inflammation mediated by TLR4. LPS-RS has two distinct mechanisms to block LPS/TLR4 signaling. One mechanism involves direct competition between acylated lipid A and hexa-acylated lipid A for binding on Myeloid differentiation-2 (MD-2) and the other mechanism involves the ability of penta-acylated lipid A: MD-2 complexes to inhibit hexa-acylated endotoxin: MD-2 complexes and TLR4 functions (Saitoh et al., 2004; Coats et al., 2005; Teghanemt et al., 2005; Visintin et al., 2005; Brubaker et al., 2015). Additional studies including those incorporating molecular approaches are needed to determine which of these TLR4-related mechanisms may be involved in the findings documented in the present study.

The present study showed that LPS-RS (but not its vehicle) when applied to the caudal medullary surface overlying the MDH can attenuate for several minutes the jaw muscle EMG activities evoked by MO application to the tooth pulp. These novel findings suggest that TLR4 processes in the MDH, the major brainstem relay site for orofacial nociceptive afferent inputs (for review, see Byers and Närhi, 1999; Iwata et al., 1999, 2011; Sessle, 2000, 2014; Chiang et al., 2011; Chichorro et al., 2017), contribute to nociceptive mechanisms related to the development of dental pain and its modulation. The present study provided additional novel findings that TLR4 is expressed in the caudal medulla as evidenced by Western Blot analysis. Interestingly, although the LPS-RS group that received the TLR4 antagonist showed reduced MO-evoked EMG activities, the expression of this receptor did not differ significantly between the naïve, LPS-RS and vehicle groups when evaluated at 2 and 15 min after MO application to the tooth pulp. There have been no previous studies in an acute pain model assessing TLR4 expression in the caudal medulla by Western Blot to allow for comparison with these findings. Thus, it is possible that the MO-evoked increase in EMG activity could be associated with TLR4 activation without increased expression, or that the time interval (15 and 2 min) after MO application to the tooth pulp for assessment of TLR4 expression may have been too short to allow for a sufficient change in expression to be detected as a significant alteration by Western Blot analysis.

While there have been no studies of TLR4 expression in the MDH, there are some relevant studies evaluating TLR4 expression elsewhere in the brainstem and in the spinal nociceptive system. A study by Ogawa et al. (2013) shows data from brainstem, but the anatomical region evaluated appears to be the rostral ventrolateral medulla and did not include the MDH. In spinal cord studies, Yan et al. (2015b) have reported that i.t. administration of Paclitaxel, a powerful anti-neoplastic drug, induces acute pain within 2 h via directly activating TLR4 and producing increased of tyrosine phosphorylation (p-TLR4) expression in the spinal dorsal horn within 1 h of its administration. At the same time, total TLR4 remained unchanged 4 h after i.v. injection of Paclitaxel (2 mg/kg). In addition, Sun et al. (2015) have evaluated TLR4 expression, albeit at a longer timeline, in the spinal dorsal horn, and reported upregulation of TLR4 expression in glial cells in the ipsilateral spinal dorsal horn in a skin/muscle incision retraction (SMIR) model; this upregulation occurred on day 5 after SMIR and was maintained until the end of the experimental period (day 20) used in their study. However, a mouse visceral pain model has been reported to be associated with no significant change in TLR4 expression in the lumbar region of the spinal cord, although there was changed TLR4 expression in the prefrontal cortex and hippocampus (Tramullas et al., 2014). Hu et al. (2018) evaluated TLR4 expression in the medulla as well as L4/L5 spinal cord at 3, 7, 11, and 14 days after nerve surgery in neuropathic models of pain (partial infraorbital nerve transection; partial sciatic ligation). These chronic pain models are quite different from our dental pain model in which we evaluated TLR4 expression at 2 and 15 min after MO application to the tooth pulp. Furthermore, the medullary region examined is not clear in the study by Hu et al. in which they found no marked changes in TLR4 expression after partial infraorbital nerve transection. Thus, the present study appears to be the first to check, by Western Blot analysis, for TLR4 expression in the region of the MDH at short time periods after noxious stimulation of peripheral tissues. Furthermore, this demonstration of TLR4 expression represents an important first step for understanding receptor signaling in MDH nociceptive processes related to TLR4 expression. It is also noteworthy that the LPS-RS dose (25 μg/10 μl) used in the LPS-RS group in the present study, although sufficient to attenuate the MO-evoked increased EMG activity, may have been too low a dose to produce significant differences between the experimental groups in TLR4 expression, or that it lacked sufficient specificity and sensitivity, as Nicotra et al. (2012) have suggested for TLR4 antibodies. The specificity and sensitivity of antibodies are important in biomarker research, and the lack of quality control tests may result in equivocal results (Signore et al., 2017). Sun et al. (2015) also used LPS-RS as a TLR4 antagonist in their SMR study, but they did not report if this antagonist had any effect on TLR4 expression. While LPS-RS is considered an LPS antagonist, its mechanism of action may not be related to TLR4 expression, but rather to the prevention of TLR4 signaling, which could also involve other molecules such as heat shock proteins (Cognasse et al., 2015; Gao and Li, 2016; Yadav and Surolia, 2019). Further studies in orofacial pain models are needed to address these possibilities. The cell types (neurons, glia) expressing TLR4 in the MDH are also worthy of future investigation since previous studies have shown that pulpal application of MO can lead to trigeminal central sensitization of MDH nociceptive neurons that is dependent on MDH microglia (Chiang et al., 2011) and that TLR4 expressed on microglia contribute to spinal cord microglial activation and central sensitization (Lehnardt et al., 2002; Olson and Miller, 2004; Guo and Schluesener, 2007; Kashima and Grueter, 2017; Bruno et al., 2018).

The present results provide evidence that tooth pulp stimulation by the TRPA1 agonist and inflammatory irritant MO induces TLR4 activation in the caudal medulla. We show for the first time that central activation of TLR4 may contribute to the mechanisms in the CNS that underlie dental nociceptive transmission. While these mechanisms likely contribute to tooth inflammatory pain in humans, additional molecular and pharmacological studies are needed to define the central role of TLR4 in dental pain and its translation to humans. An enhanced knowledge of TLR4 signaling pathways and interactions between TLR4 and other receptors promises to help guide future research aimed at developing effective drugs for controlling dental and other types of orofacial pain.

## Conclusion

This study has shown that the application of the inflammatory irritant MO to the tooth pulp can evoke a marked bilateral increase of EMG activity in the anterior digastric muscles, consistent with earlier studies. The study has also provided novel findings that TLR4 is expressed in the caudal medulla and that the application of a TLR4 antagonist to the caudal medulla can significantly attenuate the MO-evoked EMG activities but not the expression levels of TLR4 within the rat MDH. The findings suggest that TLR4 may be an important pharmacological target for the control of acute inflammatory pain involving the teeth.

## Data Availability Statement

The datasets generated for this study are available on request to the corresponding author.

## Ethics Statement

The animal study was reviewed and approved by the Animal Care Committee of the University of Toronto (protocol number #20011420) and were accomplished in accordance with the regulations of the Ontario Animal Research Act (Canada).

## Author Contributions

HF designed, conducted the experiments, analyzed the data, wrote the drafts, and finalized the manuscript. GM and YA conducted the experiments. LA-A designed, conducted and supervised the experiments, and edited the manuscript. MZ analyzed the data. S-GG and MC designed the experiments and edited the manuscript. BS designed, supervised the experiments, and edited the manuscript. All authors contributed to the article and approved the submitted version.

## Conflict of Interest

The authors declare that the research was conducted in the absence of any commercial or financial relationships that could be construed as a potential conflict of interest. The handling editor is currently organizing a Research Topic with one of the authors LA-A, and confirms the absence of any other collaboration.
